# Strengthening HR for SCM in the immunization supply chain in Nigeria through stakeholder engagement

**DOI:** 10.1186/2052-3211-7-S1-O8

**Published:** 2014-12-17

**Authors:** Ibrahim Alhaji Umar, Bervery Chawaguta

**Affiliations:** 1 National Primary Health Care Development Agency, Abuja, Nigeria; 2 UNICEF, Abuja, Nigeria

## Background

Nigeria is the most populous country in Africa with an estimated population of 176 million people and employs the largest number of health workers on the continent. The availability of human resources is at optimal levels however the human resource capacity remains a challenge in Nigeria’s health sector. National Primary Health Care Development Agency (NPHCDA) leadership has moved ahead in addressing these challenges by engaging international partners, traditional and religious leaders nationwide, and donors to be leaders and change agents in strengthening HR for SCM at all levels. This focus is aimed to improve the efficiency and effectiveness of the immunization supply chain.

## Method

The following steps were taken:

• Participating in national and state logistics working group meetings led by government, and including all key stakeholders.

• A literature review on capacity strengthening processes where NPHCDA engaged with key stakeholders at all levels, and how they relate to the programme

• Review of documented experiences and reports from deliberations during inter-agency supply chain collaboration forums and technical working groups.

• Review of reports on the 2015 Forecasting Meeting and various other meetings organised by NPHCDA in collaboration with partners.

• Review of reports on Vaccine Management Training conducted across the country.

• Recent study on Strengthening Nigeria’s Vaccines Supply Chain by McKinsey

## Results

NPHCDA leadership is determined to strengthen SCM (Supply Chain Management), by engaging development partners at all levels in order to develop strong technical leadership and enact local change. Analysis shows the benefits of strong engagement of stakeholders’ who represent different perspectives and types of expertise, in health SCM. Increased engagement of stakeholders’ in SCM is visible in inter-agency technical committees, various working groups, planning and policy decision, forecasting and procurement, cold chain management, logistics, communication for development, social mobilization, monitoring and evaluation, data management, and service. This engagement has included secondments of supply chain specialists to work fulltime with NPHCDA HR for SCM to transfer knowledge, provide training, and technical assistance in key areas of collaboration.

## Discussion

NPHCDA should continue engaging partners for SCM, and play the key leadership role and leverage partner support for advocacy at all levels in order to drive sustainable change in the supply chain. Engaging key partners representing different perspectives and expertise, to develop human resources capacity for SCM at all levels will help in building consensus and promote national and local ownership, leadership consistent and ensure a sustainable exist strategy. NPHCDA should set the agenda and support dialogues of accountability at all levels through its 2013 Accountability framework.

## Lessons learned

NPHCDA’s increased collaboration with partners at all levels, has helped in increasing the immunization coverage by 50% over the past 3 years, because of strong leadership and focus, oversight from stakeholders and skills development of NPHCDA HR for SCM. Stakeholders have also played a key role in advocating for change at all levels and SCM is now acknowledged as a key driver for successful programme implementation.

